# 3D morphology of the human hepatic ferritin mineral core: New evidence for a subunit structure revealed by single particle analysis of HAADF-STEM images

**DOI:** 10.1016/j.jsb.2008.12.001

**Published:** 2009-04

**Authors:** Ying-Hsi Pan, Kasim Sader, Jonathan J. Powell, Andrew Bleloch, Mhairi Gass, John Trinick, Alice Warley, Andy Li, Rik Brydson, Andy Brown

**Affiliations:** a Institute for Materials Research, University of Leeds, Leeds LS2 9JT, UK; b SuperSTEM, Daresbury Laboratories, Warrington WA4 4AD, UK; c Institute for Molecular and Cellular Biology, University of Leeds, Leeds LS2 9JT, UK; d MRC Human Nutrition Research, Cambridge, UK; e CUI, King’s College London, London SE1 1UL, UK

**Keywords:** Ferritin, Ferritin core subunit structure, Scanning transmission electron microscopy (STEM), Electron fluence, Electron energy loss spectroscopy (EELS)

## Abstract

Ferritin, the major iron storage protein, has dual functions; it sequesters redox activity of intracellular iron and facilitates iron turn-over. Here we present high angle annular dark field (HAADF) images from individual hepatic ferritin cores within tissue sections, these images were obtained using spherical aberration corrected scanning transmission electron microscopy (STEM) under controlled electron fluence. HAADF images of the cores suggest a cubic morphology and a polycrystalline (ferrihydrite) subunit structure that is not evident in equivalent bright field images. By calibrating contrast levels in the HAADF images using quantitative electron energy loss spectroscopy, we have estimated the absolute iron content in any one core, and produced a three dimensional reconstruction of the average core morphology. The core is composed of up to eight subunits, consistent with the eight channels in the protein shell that deliver iron to the central cavity. We find no evidence of a crystallographic orientation relationship between core subunits. Our results confirm that the ferritin protein shell acts as a template for core morphology and within the core, small (∼2 nm), surface-disordered ferrihydrite subunits connect to leave a low density centre and a high surface area that would allow rapid turn-over of iron in biological systems.

## 1 Introduction

Iron is an essential element for biological processes such as oxygen transport, cellular respiration, and DNA synthesis. This is because of its ability to readily cycle between the ferrous (Fe^2+^) and ferric (Fe^3+^) states. At pH 7 Fe^2+^ is soluble in aqueous solution and so is bio-available, but due to its capacity to generate the hydroxyl radical (via Fenton chemistry) can be damaging to cellular components. Conversely, Fe^3+^ is insoluble in aqueous solution at circum-neutral pH and is thermodynamically stable under oxidising conditions, therefore is not readily bio-available and is non-toxic. Cells and organisms have developed methods of overcoming the toxicity and poor availability of iron: one well-documented aspect of this is the storage of iron within the ferritin molecule in an accessible and non-toxic mineral form. Ferritin is the primary storage molecule in most living cells throughout the animal, plant, and microbial kingdoms (Chasteen and Harrison, 1999).

During the daily human iron cycle excess iron is temporarily stored by intracellular ferritin molecules. Disorders in the human metabolism and storage of iron include haemochromatosis (Barton and Bertoli, 1996) and Friedreich’s ataxia (although this particular disorder is associated with the iron storage protein, mitochondrial frataxin, Karlberg et al., 2006). Problems with the storage of iron are also increasingly being recognised as important in neurodegenerative disorders including Alzheimer’s disease (Collingwood and Dobson, 2006) making it pertinent to understand the exact structure and morphology of the mineral form in which ferritin sequesters iron.

The ferritin molecule is a 24 subunit oligomer of mass ∼500 kDa with a combination of heavy (H) and light (L) subunits that form a hollow protein structure ∼12 nm in diameter with cubic point group symmetry 432, and an ∼8 nm diameter central cavity in which the iron is stored (Harrison and Arosio, 1996). There are channels along both the 3- and 4-fold symmetry axes of the shell of the molecule which allow Fe^2+^ ions to enter and exit the central cavity. The H subunits of the shell contain a hydrophilic region centrally located within an internal structure that houses dinuclear centres that catalyse the oxidation of Fe^2+^ ions to Fe^3+^ during storage (the so-called ferrioxidase centres; Chasteen and Harrison, 1999). In the L subunits this central region varies more across ferritin types and is associated with enhanced subunit stability (Hempstead et al., 1997). Additionally, both subunits have a group of glutamates on their inner surface that for the L subunit are associated with mineralisation on the inner surface of the protein shell *in vitro*, whilst it has been shown that this ‘nucleation site’ in human H-chain ferritin is not required for mineralisation of the iron core (Bou-Abdallah et al., 2004). The complementary functions of ferroxidation and mineralisation associated with the two subunits are utilised in hetropolymer proteins, since varying the mixture of subunits affects the amount of iron incorporation and mineralisation in the core, reaching a maximum with only 18–30% H subunit content (Levi et al., 1994).

Iron is stored in the ferritin core in a complex similar to the hydrous ferric oxide mineral, ferrihydrite. Despite numerous studies both the exact structure and morphology of this core remain controversial. Unlabelled ferritin molecules can be observed by analytical microscopy in any cell type, providing a sufficient amount of iron is present. There is a long history of the electron microscopic study of ferritin, with the mineral core first being identified by transmission electron microscopy (Farrant, 1954). In the 1960s the core was proposed to have substructure consisting of mineral units arranged at the vertices of an octahedron (Bessis and Breton-Gorius, 1960; Muir, 1960) although the validity of this model has been disputed since many of the core projections could not be explained by any possible orientation of the model (Gachet and Theiry, 1964). Furthermore, the observation by Haydon (1969) that apparent substructure in the cores could result from a phase contrast granularity artefact arising from the background of a defocused carbon support film overlaying the image of a mineral core when recorded in bright field transmission electron microscopy (TEM) cast much doubt on the ‘subunit’ hypothesis. In a response to Haydon’s paper Harrison and Hoy (1970) did not dispute the phase contrast induced imaging artefact but pointed out that core-substructure may exist since ‘Haydon interprets the internal structure of the core as a random distribution of unit cells’ and that this was incompatible with the available X-ray diffraction evidence (Harrison et al., 1967) which shows that the core is crystalline and is composed of ‘either a single crystallite or possibly in some molecules of a small number of crystallites’. Haggis (1970) put forward that the then emerging technique of dark field Scanning (S)TEM would settle the debate because of the absence of phase contrast effects in such images. A number of studies have used dark field STEM (Ohtsuki et al., 1979; Isaacson and Ohtsuki, 1980) and dark field TEM (Massover, 1993) and have observed substructure in ferritin cores although it was not considered to be regular or correlated to the symmetry of the protein shell. Cowley et al. (2000), suggested that substructure observed in previous dark field STEM images of ferritin was due to specimen mass variations unrelated to the mineral core. Additionally, Massover (Massover and Adrian, 1986; Massover, 1993) observed substructure using bright field cryo-TEM of vitrified ferritin, though he dismissed this as an artefact due to Fresnel diffraction.

In this work we use the compositionally sensitive high angle annular dark field imaging mode of the STEM, free of both phase contrast and diffraction contrast, to visualise ferritin molecules *in situ* in sections of a biopsy sample of human liver from a patient with haemochromatosis. We used a spherical aberration corrected STEM capable of the routine formation of a 0.13 nm (FWHM) electron probe, that allows HAADF imaging at atomic resolution (Bleloch and Lupini, 2004). This enabled us to show an increased signal to noise in HAADF images of the mineral core compared to simultaneously acquired BF STEM images and hence demonstrate that there is clear substructure to ferritin cores when the images are in focus. Using electron energy loss spectroscopy and progressively increasing electron doses we were able to assess any changes in the mineral core that are induced by the application of the electron beam. Finally, we used single particle image processing techniques to show that averages of HAADF images from many cores with common projection directions provide indisputable evidence for a regular subunit structure to the core.

## 2 Methods

### 2.1 Scanning transmission electron microscopy

Thin sections of a liver biopsy from a patient with hereditary haemochromatosis type 2, juvenile haemochromatosis, were prepared as described by Pan et al. (2006). Ethical permission and patient consent were obtained. We concentrate on the analysis of cytosolic ferritin although first we assessed the electron beam sensitivity of iron stored in clusters of lysosomal haemosiderin molecules. The biopsy sections were analysed using a VG-HB501 STEM (SuperSTEM) situated at Daresbury Laboratories, UK. The instrument is equipped with a cold field emission filament operating at 100 kV ± 0.3 eV, a Gatan Enfina electron energy loss spectrometer with a total spectral resolution of 0.5 eV, a CCD camera and DigitalMicrograph 3.9.3 with Gatan Microscopy Suite (GMS) 1.4.3. The SuperSTEM combines a VG base microscope fitted with a Nion C_s_ corrector (14 dipoles, 7 quadrupoles and 3 octupoles). The electron beam convergence semi-angle is 24 mrad and the collection semi-angle for EELS is 19 mrad. The HAADF detector gathers electrons scattered through a semi-angle range of 70–210 mrad, namely those electrons that are Rutherford scattered. The bright field detector gathers electrons scattered through up to 8 mrad. The minimum focused probe diameter obtained with this microscope is 0.91 Å with a measured current at the specimen of 100 pA. High resolution HAADF images of 1024 × 1024 pixels were acquired digitally with a Gatan Digiscan unit from the photomultiplier of a modified Vacuum Generators HAADF detector.

### 2.2 Electron energy loss (EEL) spectrum imaging under progressively increasing electron dose

The stability of the ferritin core was assessed under progressively increasing electron fluence (electrons/nm^2^) and therefore increasing quantity of energy absorbed by the specimen (electron dose) similar to that described previously (Pan et al., 2006). The valence and oxygen co-ordination of the iron in the mineral cores was monitored as a function of electron fluence by quantifying the energy-loss near-edge structure (ELNES) of the Fe *L*_2,3_-ionisation edge measured using EELS. Fe *L*_2,3_-edges from a single specimen area were recorded at progressively increasing electron fluence and then the shape of the edges quantified by fitting four reference spectra that had been obtained from minerals with just Fe^2+^ or Fe^3+^, either tetrahedrally or octahedrally co-ordinated with oxygen, to the experimental edge using a non linear least-square (NLLS) fitting routine.

Fe *L*_2,3_-core edge EEL spectra from haemosiderin were acquired by using the spectrum imaging technique. A defocused electron probe was used to scan (i.e., irradiate) a small area (effectively the defocused probe being moved very little) within a haemosiderin cluster in the liver biopsy. By measuring the probe current and then gradually focusing the probe, the accumulated electron fluence (and current density) could be varied. The minimum accumulated electron fluence used for collecting *L*_2,3_-edges with enough signal for fitting the energy-loss near-edge structure (ELNES) was 6 × 10^3^ electrons nm^−2^ (probe diameter = 653 nm) and this was increased up to an accumulated electron fluence of 6 × 10^7^ electrons nm^−2^ (probe diameter = 86 nm). Up to the accumulated electron fluence of 10^6^ electrons nm^−2^, a very small current density was used (300 A/m^2^; probe diameter = 653 nm) but the current density was increased (to 1.7 × 10^4^ A/m^2^) when the electron probe was changed to 86 nm in diameter.

Single cytosolic ferritin cores in the haemochromatosis liver biopsies were also examined in terms of their ELNES. Since there is much less iron in a single ferritin core compared to a haemosiderin cluster, a focused electron probe (0.13 nm in diameter) was used and this probe was manually moved around an individual core to minimise the dose applied to any one part of the core. For these analyses the electron dose cannot be as low as when a defocused probe is used. For the ELNES analysis of single cores, the estimated accumulated electron fluence (assuming even illumination of a core) ranged from 1.1 × 10^7^ to 1.5 × 10^8^ electrons nm^−2^ in the haemochromatosis liver biopsy (Pan, 2007). It should be noted that although a similarly focused probe was used for HAADF imaging (routinely 0.13 nm in diameter but at optimum 0.091 nm in diameter, both giving a current density ∼10^10^ A/m^2^) the dwell time was significantly shorter than that used for haemosiderin or ferritin Fe *L*_2,3_-ELNES acquisition (19.5 μs for imaging versus 10 s for EELS) resulting in an accumulated electron fluence of ∼10^6^ electrons nm^−2^ for the HAADF imaging.

Once acquired, the Fe *L*_2,3_-edges were energy calibrated, background stripped using a power law background extrapolation, low-loss deconvoluted and then area normalised by using an energy integration window of 28 eV that included the two iron white lines (from 702–730 eV). Fe-valence and Fe–O co-ordination were then determined by fitting reference spectra to the measured Fe *L*_3_-edges (702–722 eV) this procedure was originally developed by Garvie and Buseck (1998). The spectra obtained from hepatic ferritin were fitted with four reference-mineral spectra recorded on the STEM under the same experimental conditions. The four minerals used to obtain reference spectra were: haematite taken to be representative of Fe^3+^ in octahedral co-ordination with oxygen; Fe-Orthoclase representative of Fe^3+^ in tetrahedral co-ordination; Hedenbergite representative of Fe^2+^ in octahedral co-ordination; and Hercynite representative of Fe^2+^ in tetrahedral co-ordination (Pan, 2007). The fitting was carried out using a non linear least squares fitting routine within the IGOR Pro 5.0 software and, each of the fitting coefficients for the reference spectra was constrained to be ⩾0, small energy shifts (±0.3 eV) were allowed, and *χ*^2^ was reported so that an estimated error for each coefficient could be calculated to indicate the quality of fit (Pan, 2007). As a check we also estimated the Fe-valence by taking the ratios of the integrated *L*_3_- to *L*_2_-edge intensities following the universal curve procedure of van Aken and Liebscher (2002).

### 2.3 EEL spectrum imaging and absolute quantification of iron levels

The absolute quantification of iron levels as measured in the EEL spectrum image of individual cores was undertaken using the procedure described in Pan et al. (2008). This involved the following steps: (a) the spectrum image was centred around an individual core and set to be 10 pixels by 10 pixels (1 nm/pixel), a spectrum of the Fe *L*_2,3_-ionisation edge (702–730 eV energy loss) was acquired for every pixel with a spectral energy dispersion of 0.2 eV/channel and acquisition time of 1 s; (b) low-loss EEL spectra (0–200 eV energy loss) were acquired from the same pixels using the same energy dispersion but with a shorter acquisition time (0.01 s/pixel) and also with the spectrometer strongly defocused to maintain the same probe current at the specimen as used for the Fe *L*_2,3_-edge acquisition but without saturation of the EELS detector; (c) the total number of iron atoms in each ferritin core was then estimated from each pair of EEL spectra (core-edge and low-loss) taken at each pixel in the imaging box by applying Eq. (1) shown below and summing up the number of iron atoms in each pixel (*N*).

(1) $$N=\frac{I_{\text{Fe}}(\beta \text{,}\;\Delta \text{,}\;E_{0})}{I_{\text{l}}(\beta \text{,}\;\Delta \text{,}\;E_{0})\sigma_{\text{Fe}}(\beta \text{,}\;\Delta \text{,}\;E_{0})}$$

where *N* is the areal density (atoms/nm^2^) of the atoms giving rise to the Fe *L*_2,3_-ionisation edge, *I*_l_ is the integrated number of counts in the low-loss spectrum, *I*_Fe_ is the integrated number of counts under the background subtracted Fe *L*_2,3_-edge, σ*_Fe_* is the partial cross-section for Fe-2*p*-ionisation for the experimental incident beam energy (*E*_0_), effective collection semi-angle (*β*) and edge integration window (*Δ*).

The validity of this method was tested using maghemite, a well-characterised iron oxide reference mineral (details in Pan et al., 2008). Four synthetic maghemite particles that had been imaged with a projection and crystal orientation consistent with cubeoctahedral morphology were used. EELS measurements were made and iron content in the spectra were compared with that estimated using the known composition, shape and projected dimensions of the particles. This procedure leads to an estimated uncertainty of 20% in the EELS absolute quantification process. Taking this validated EELS method, 16 cytosolic ferritin molecule cores with a range of HAADF image intensities, and hence differing iron contents, were measured for calibration of their respective HAADF image intensities. The most iron-filled core of the 16 was estimated to contain 2100 ± 400 iron atoms and the least iron-filled core to contain 320 ± 60 iron atoms.

### 2.4 Single particle analysis-three dimensional reconstruction

Image processing and 3D reconstruction work was carried out on 133 STEM-HAADF images that had been acquired at 100,000× magnification. From these the images of individual particles were windowed using the BOXER software (Ludtke et al., 1999) with a selection box 100 pixels by 100 pixels (0.146 nm/pixel). In this selection procedure, only isolated cytosolic ferritin cores were chosen and the particle images normalised to the background using a 10-pixel ring from around the outer edge of the image of the core (Sorzano et al., 2004) and were band pass filtered to remove noise and any relatively long range intensity variation: this procedure was implemented in the SPIDER image processing software. The processed particle images were first centred and then aligned and classified into 14 class averages (based on core shape) using EMAN image processing software. Several of the class averages were used to generate a preliminary 3D model (model (a) in Fig. 3d) by using the common lines of the Fourier transforms of the class averages (a cross-common lines method implemented in EMAN), without imposing any symmetry.

To select a subset of ferritin cores with similar iron loading, for a more representative 3D reconstruction, the HAADF image intensity of 16 separate molecule cores was calibrated against their iron content measured using the EELS elemental iron quantification described above and in Pan et al. (2008). The calibration factor was obtained by linear correlation of the EELS iron quantification method to the normalised HAADF image intensity (accounting for the estimated uncertainty of ±20% in the EELS data points; *N*(Fe) = 0.000136 × normalised image intensity + 0.00168). On application of this calibration relationship the whole particle image data set was divided into 10 groups according to the respective iron-filling of each core. [It should be noted that the distribution shown in Pan et al. (2008) was erroneously obtained using a linear correlation (*N*(Fe) = 0.000207 × [normalised image intensity] − 0.07) that did not account for the estimated uncertainty of the data points]. The three most populated groups of cores (750 in total, each containing 1100–1850 iron atoms) were used for the following reconstruction work. The processed particle images were first centred and then aligned and classified into 18 class averages using EMAN image processing software. Three different initial starting models related to the preliminary reconstruction (the original itself, eight spheres in a simple cubic arrangement and a sphere) were then used to reconstruct 3D models of a core containing 1100–1850 iron atoms.

## 3 Results

### 3.1 The effect of accumulated electron dose on the structure of the ferritin core

**Fig. 1:**
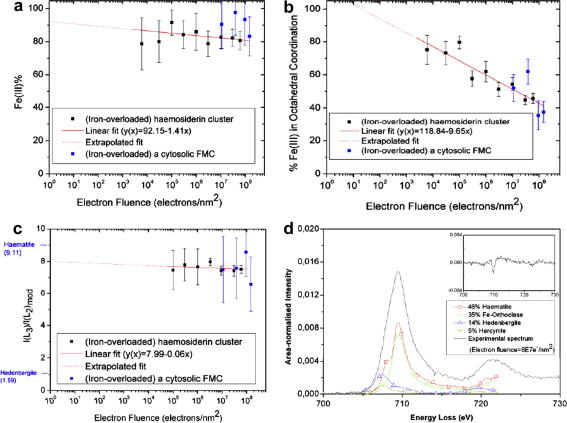
The effect of accumulated electron fluence on (a) the valence and (b) the oxygen co-ordination of iron in haemosiderin and ferritin mineral cores (HMC and FMC respectively) as measured by fitting reference spectra to Fe *L*_2,3_-ionisation EELS edges recorded in the STEM. (c) The change in valence with progressively increasing electron fluence as measured by taking Fe *L*_3_ to *L*_2_- ratios from the same spectra used for a and b, following the method of van Aken and Liebscher (2002). (d) The highest fluence haemosiderin Fe *L*_2,3_-ionisation EELS edge with the four fitting reference spectra shown underneath and their intensity scaled by the fitting coefficients such that when summed they produce the best fit to the mixed valence spectrum (inset is the residual after fitting).

The effect of electron fluence on the mineral structure of the ferritin core was monitored by quantifying the energy-loss near-edge structure (ELNES) of the Fe *L*_2,3_-ionisation edges measured from the core using STEM–EELS (Fig. 1). Unlike the parallel illumination of the 200 keV TEM used previously (Pan et al., 2006), the focused yet scanned STEM probe of 100 keV electrons produced some changes in iron–oxygen co-ordination but almost no change in iron valence over a range of electron doses (100 keV electron fluence from 6.0 × 10^3^ to 1.6 × 10^7^ electrons nm^−2^ at current densities from 300 to 1.7 × 10^4^ A/m^2^). As expected, at the lowest dose, the iron in all cores was found to be composed of at least 90% Fe^3+^ in a ferrihydrite-like structure with at least 80% of it being octahedrally co-ordinated. Whilst at the highest dose octahedral co-ordination decreased to approximately 50% of its initial level.

### 3.2 High angle annular dark field (HAADF) STEM images of the ferritin core

**Fig. 2:**
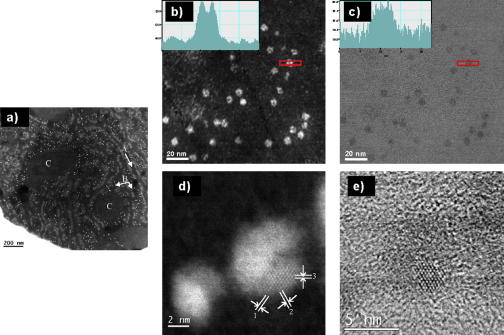
STEM-HAADF images of ferritin molecule cores within thin sections of fixed and unstained tissue. (a) Ferritin cores located in a hereditary haemochromatosis human liver biopsy. Strongly scattering iron-rich cores are visible as bright spots in the image and occur throughout the cytoplasm. Some cellular structures can be identified: A: endoplasmic reticulum; B: ribosome; C: organelle (probably mitochondrion). (b) Intermediate magnification HAADF image within the tissue section showing clear and regular subunit structure to the cytosolic ferritin cores with a line profile of the signal to background across a core inset. (c) Bright field STEM image of the same area as (b); note the significant decrease in signal to background of the inset line profile across a core compared to the HAADF image. (d) High magnification image of cytosolic ferritin cores from the same biopsy showing atomic lattice resolution of a subunit within a core. While other parts of the core may also be crystalline, only the bottom-right corner of the core is oriented along a crystallographic zone axis such that iron atom columns are resolvable/visible in the image. Note the non-facetted nature of the core edges. The lattice *d*-spacings (0.273 nm for spacing 1 and 0.272 nm for spacing 2, and 0.276 nm for spacing 3) and angles between the spacings (58.7° and 60.2°) are consistent with a ferrihydrite crystal structure (Drits et al., 1993). (e) Corresponding bright field STEM image of the core shown in (d) here the image is dominated by phase contrast effects such as the granularity in the embedded tissue surrounding the core and the lattice fringes in the subunit of the core that is lying on a crystallographic zone axis. i.e., the size and shape of the core is not clear here in comparison to the HAADF image because the parts of the core that are not lying on a crystallographic zone axis are partially obscured.

**Fig. 3:**
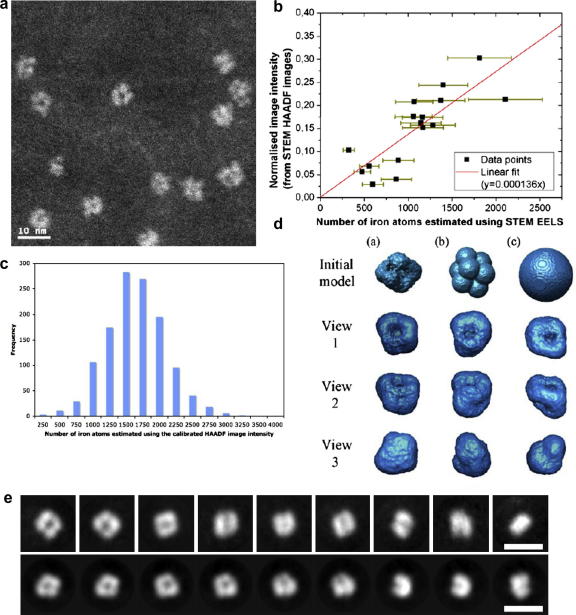
Summary of the single particle reconstruction procedure for an average hepatic ferritin molecule core with an estimated iron loading of between 1100–1850 iron atoms. (a) An example of one of 133 HAADF images containing multiple ferritin cores, used for the reconstruction. (b) Absolute quantification of the iron atom content of a core using EELS correlates linearly with the normalised HAADF image intensity for a set of 16 ferritin cores with a range of different iron loadings. (c) Distribution of the iron content of 1241 ferritin cores in a tissue section showing a normal distribution with a mean of 1500 ± 300 iron atoms (upper and lower limits are 4,000 and 200 iron atoms). (d) Selected views of the initial models and final reconstructions (view 1–3) of a core developed from the 750 ferritin cores each with an iron atom content in the most populated range of between 1100–1850; despite different initial models a cubic-like subunit structure with a low density centre is evident in all of the final reconstructions. (e) The top row shows 750 core images all bandpass filtered, aligned and classified using the EMAN program into 9 of the 18 projection classes. The bottom row shows 9 re-projections, at 12° spacing, of the final reconstruction developed from initial model (a). The re-projections show strong correlation with the raw shape classes. In both cases the scale bar is 10 nm.

**Fig. 4:**
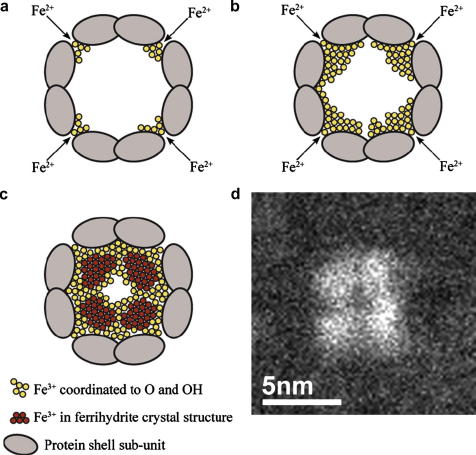
Schematic cross-section (viewing direction: parallel to one of the four-fold symmetry channels in the protein shell) of a hepatic ferritin core depicting our proposed formation mechanism. This is a modification of a schematic of core formation by Lewin et al. (2005). (a) Early stage of iron deposition in the ferritin central cavity. The sites near the ends of the three-fold symmetry iron entry channels (where the protein shell subunits, shown as grey lobes, have specific oxidation sites) are favourable for the incoming Fe^2+^ to deposit and be oxidised. The yellow circles represent oxidised iron (Fe^3+^). (b) As the iron cellular concentration increases, more Fe^2+^ is shuffled into the molecule and may rapidly deposit and oxidise on the surface of any existing Fe^3+^ deposits near the entry channels; consequently, core subunits are formed. (c) With higher iron-filling, a cubic-like core structure with eight subunits (only four of which are shown) develops. Oxidation of further incoming Fe^2+^, results in the early deposited Fe^3+^ diffusing inwards forming closely packed crystalline structures of ferrihydrite (dark red circles in contrast to the loosely packed Fe^3+^ (yellow circles)), the atomic structure of such a subunit structure is seen experimentally in Fig. 2d. The surface of each core subunit is disordered facilitating dynamic load and release activities consistent with the ‘last-in first-out’ hypothesis (Hoy et al., 1974). (d) An example of a commonly observed HAADF image of a single ferritin core of similar iron loading and lying in a similar orientation to the schematic; the four-fold symmetry arrangement of the subunits and a low density central region are clearly evident.

Previously we have shown that staining with uranyl acetate and lead citrate normally used to generate image contrast in the TEM changes the chemical state of the iron within tissue sections by promoting oxidation (Brown et al., 2003). As we wanted to best preserve the local chemistry (Dobson and Grassi, 1996), only chemically-fixed but unstained biopsies were examined in this work. In the HAADF or Z-contrast images of these biopsies, cytosolic ferritin cores appear bright due to the relatively high atomic number of the iron atoms in the core compared with that of the organic material in the surrounding tissue (Fig. 2a). Although the biopsy sections were unstained the inclusion of osmium tetroxide in the preparation procedure enabled visualisation of some of the main cellular structure in the HAADF images allowing location of the relative position of the cores within the cell (Fig. 2a). This is a major advantage over standard TEM of such unstained sections where identification of the core location is difficult using bright field imaging. HAADF images of cytosolic hepatic ferritin molecules showed very obvious subunit structure to the cores, a square envelope to core projections was frequently observed and a low density centre to the core projections was often evident (Figs. 2b, 3a and 4d). The images suggest that cores are composed of discrete subunits assembled in a cubic array and this will be discussed further following the 3D morphological analysis. There is a high signal to background ratio in these HAADF images compared to bright field STEM images of the same areas (for example, compare Fig. 2b and c or Fig. 2d and e) and unlike bright field imaging there is no superposition of the phase contrast induced granularity of the supporting tissue (Haydon, 1969). Fresnel fringes present at the large defoci employed to generate sufficient contrast in TEM (Massover and Adrian, 1986) are also absent in STEM-HAADF images.

High magnification HAADF images of cytosolic molecules confirmed the polycrystallinity of the cores (Fig. 2d and e) and whilst this finding is certainly not novel the additional signal to background in the aberration corrected HAADF images clearly shows within the limits of the applied electron dose that the crystalline regions have a disordered iron-rich surface structure (Fig. 2d). This detail is not as apparent in bright field images (Fig. 2e) or previous HAADF-STEM images (Isaacson and Ohtsuki, 1980; Cowley et al., 2000).

### 3.3 Single particle reconstruction of the ferritin core morphology

Single particle analysis was employed to reconstruct the three dimensional (3D) structure of an average core from the HAADF projection images of 1241 hepatic ferritin cores recorded in this study. This analysis is designed for identical objects with random orientation in thin layers and is commonly applied to low dose electron cryo-TEM images (Ludtke et al., 1999). We applied the technique in this case because the HAADF images suggested a regular subunit structure to the cores (Figs. 2b and 3a). The HAADF image contrast shows an approximate sensitivity to the atomic number of the constituent atoms of the imaged material to the power 1.7 and/or to the thickness of the specimen (Pennycook et al., 2000) (the specimen thickness in this case, is to a first approximation, uniform on the scale of individual ferritin molecules as it was prepared using an ultra microtome). However, as ferritin cores in human tissues contain different amounts of iron, it is not valid simply to reconstruct an average core based on every core image (as was done for our initial core reconstruction which suggested a correlation with the cubic point group symmetry of the protein shell (Fig. 3d, initial model (a) with 7 nm high pass filtering)). Therefore we have used the HAADF image intensity calibration outlined in the Section 2 (Fig. 3b) to pre-classify the iron content of each of the 1241 individual cores imaged, to obtain a distribution of iron loadings showing that the number of iron atoms per core ranged from 200 to 4000 (Fig. 3c). The three final reconstructions derived from three different starting models and without imposing any symmetry were produced from images (with 10 nm high pass filtering) of cytosolic ferritin cores with the most common iron loading in the iron-overloaded human liver tissue of 1100–1850 iron atoms. All three starting models produced reconstructions that converge to very similar structures, each with 2 nm resolution given by the 0.5 cut-off of the Fourier shell correlation curve (Fig. 3d). Back projections of specific orientations of the reconstructed 3D model produced from initial starting model (a) (Fig. 3d) show consistency with the aligned classes (Fig. 3e) as well as the observed projections of individual cores in raw images, (such as Figs. 3a and 4d). The set of 18 class averages of the cores (Fig. 3e) exhibit specific projections suggestive of the cubic symmetry of the protein shell of the molecule (Ford et al., 1984; Harrison and Arosio, 1996). Our images and 3D reconstruction of the average hepatic ferritin core suggest it has eight subunits in an arrangement that reflects the cubic symmetry of the protein shell of the molecule and its eight, three-fold symmetry entry channels for Fe^2+^. The fact that the subunits do not meet in the centre of the core is consistent with a core containing up to only ∼40% of its maximum possible loading of 4,500 iron atoms (Ford et al., 1984; Harrison and Arosio, 1996). The low density centre is also evident in a slightly lower resolution voxel projection of a volume reconstruction of a STEM-HAADF tilt series (electron tomography) taken from the same biopsy sections as those used in this study (Weyland, 2005).

## 4 Discussion

We have used STEM-EELS to monitor the effect of high energy electrons on haemosiderin mineral cores within thin sections of human liver, fixed immediately after biopsy, by quantifying the energy-loss near-edge structure (ELNES) of the Fe *L*_2,3_-ionisation edges measured using STEM-EELS (Fig. 1). We have previously shown using TEM (Pan et al., 2006), that the haemosiderin mineral structure alters with increasing electron dose, here using STEM we show that the core alters from an Fe^3+^ bearing mineral in which the Fe is > 80% octahedrally co-ordinated at the lowest dose to a Fe^3+^ bearing mineral in which the Fe is ∼50% octahedrally co-ordinated at the highest electron dose. Since haemosiderin is considered to be a degraded form of ferritin (Andrews et al., 1987) one presumes such electron dose behaviour will also be applicable to ferritin mineral cores. This is to some extent confirmed here because the relatively high electron dose Fe *L*_2,3_-ELNES measurements taken from individual ferritin molecules are consistent with the haemosiderin data (Fig. 1). Furthermore, our ELNES analysis concurs with extended X-ray absorption fine structure analysis (EXAFS) of the iron *K*-edge of ferritin extracted from horse spleen and haemosiderin extracted from the livers of humans with primary haemochromatosis which show that, on average, the environment around each iron atom within *either* structure is similar and consists of approximately six nearest neighbour oxygen atoms (Mackle et al., 1991). The EXAFS data indicate that the *extracted* haemosiderin is amorphous whereas selected area electron diffraction recorded in the TEM from the haemosiderin *within* the tissue sections has a polycrystalline, ferrihydrite-like, structure (Pan et al., 2006). More importantly, previous analysis of individual ferritin molecules using electron nanodiffraction (END) (Cowley et al., 2000) combined with atomic lattice imaging (Quintana et al., 2004) and atomic lattice imaging combined with EELS (Quintana et al., 2000) applied both in STEM and TEM have suggested that individual cores are composed of one of several possible inorganic phases, ranging from ferrihydrite (FeOOH→Fe_5_HO_8_·4H_2_O) (Towe and Bradley, 1967) in the majority of cores, through to magnetite (Fe_3_O_4_) in a minority. Since magnetite contains both Fe^3+^ and Fe^2+^ ions, under certain conditions, redox cycling could occur even within ferritin itself. Whilst the possibility that the high energy electron beam might itself induce transformation of the iron oxide phases was acknowledged it was not carefully precluded in any of these studies. We estimate that the minimum electron fluence applied to ferritin cores during STEM-nanodiffraction based on the procedure reported in Cowley et al., (5 nm diameter probe, dwell time of 0.033 s and assuming a beam current of 100 pA) to be ∼10^6^ electrons nm^−2^. In the 100 kV STEM, this fluence is high enough to convert significant amounts of octahedrally co-ordinated iron in a mineral core to tetrahedral co-ordination (Fig. 1) which could lead to the identification of an iron oxide with a spinel structure such as that of maghemite/magnetite. In the 200 kV TEM this fluence (we estimate a similar flux is applied during atomic lattice imaging such as that used by Quintana et al., 2000) is enough to produce co-ordination and valence changes (Pan et al., 2006) that could lead to the identification of magnetite in individual cores.

Galvez et al. (2008) conducted STEM-EELS *across* an individual, chemically reduced, ferritin core extracted from horse spleen and using Fe *L*_2,3_-ELNES, identified Fe^2+^ at the surface of the core. Based on the STEM-EELS procedure reported by Galvez et al., (0.5 nm diameter probe, 1 s dwell time and assuming a 100 pA probe current), we estimate that they applied a fluence of ∼3 × 10^9^ electrons nm^−2^ for each spectrum recorded and therefore from our current results (Fig. 1) would expect the production of some Fe^2+^ at this fluence. Galvez et al. (2008) corroborated their findings by identifying a significant component of magnetite in the same fractions of horse spleen ferritin using lower dose X-ray absorption spectroscopy (XANES) of the Fe *K-*edge. They did so by fitting with only three reference spectra that included magnetite, haematite and ferrihydrite rather than systematically identifying the fractions of iron in different co-ordinations and valences as we have done here. The evidence obtained from XANES analysis suggests the persistence of Fe^2+^ inside the protein coat of horse spleen ferritin during experimental reconstitution of cores, with Fe^2+^ persisting for ⩾16 h in air (Rohrer et al., 1987, 1989), however, it is unclear whether this persistence occurs during any chemical reduction used to remove iron from cores of extracted ferritin, such as that applied by Galvez et al. (2008) whose ferritin solutions were dialysed for 4 days at 4 °C after iron removal. Magnetic measurements of liver iron deposits in tissue from an iron-overloaded rat model show no contribution from a magnetite like phase (Gutiérrez et al., 2006). To re-iterate, our results, which accurately account for the effects of electron fluence, suggest that the pristine human hepatic ferritin within rapidly fixed and unstained tissue sections has a mineral core similar in phase to ferrihydrite, containing only Fe^3+^, the majority of which is in octahedral co-ordination with the surrounding oxygen ions.

STEM-HAADF imaging demonstrates that the mineral cores of hepatic ferritin exhibit a cubic external morphology and have a substructure that frequently results in a low density centre to the core (Fig. 2). These findings are not as apparent in bright field STEM imaging and this is consistent with the bright field TEM imaging discussed by Haydon (1969). The electron fluence applied to collect the 100,000 times magnification images was 9 × 10^5^ electrons nm^−2^ (0.13 nm diameter probe, 19.5 μs dwell time and 100 pA probe current) but 2.3 × 10^7^ electrons nm^−2^ for the 500,000 times magnification images (Fig. 2d). Isaacson and Ohtsuki (1980) reported only a 5% mass loss from STEM dark field images of extracted horse spleen ferritin cores taken at electron fluence up to 5 × 10^5^ electrons nm^−2^ (with incident energies of 30–40 keV) and argued that the bright contrast in the STEM dark field images of ferritin correlates with the iron in the mineral core. Shuman and Somlyo (1982) and Pan (2007) have confirmed the latter using energy-filtered TEM and STEM-EDX linescans, respectively. Consequently, we believe that the dose applied to obtain the HAADF images of ferritin presented in this study (recorded at 100,000 times, with 100 keV electrons at 9 × 10^5^ electrons nm^−2^) is low enough that the images are likely to reflect the true morphological structure of the mineral cores within tissue rather than an artefact induced by the electron beam. This is especially so since the work has been undertaken on ferritin *in situ* within unstained sections obtained from tissue that was fixed immediately after biopsy rather than on extracted or reconstituted ferritin. Furthermore Cooper et al. (1988) cited in Iancu (1992) shows that immunogold labelled anti-ferritin antibody recognises the ferritin in the cytoplasm of liver cells from haemochromatosis patients suggesting that normal protein structure is maintained.

Isaacson and Ohtsuki (1980) used an inelastic-elastic difference-based STEM signal to demonstrate that the central region of extracted horse spleen cores are iron deficient relative to the mantle and also noted ‘apparent substructure in the core’. Our (HAADF-STEM) results show similar features present in the human hepatic ferritin core. To quantify this substructure we recorded images of over 1000 molecules within a tissue section: the background-normalised, aligned and class average of these images support a regular core-substructure in the absence of phase contrast or diffraction imaging artefacts (Fig. 3). The class averages suggest a cubic morphology to the core (Fig. 3e). Our 3D reconstructions of an average ferritin core are consistent with such a structure (Fig. 3d), although the combination of particles with varying numbers of iron atoms (1100–1850) as well as possible variations in subunit organisation degrade the resolution of the model. Nonetheless, a cubic morphology is consistent with angular-precession X-ray diffraction studies of single crystals of the most fully loaded fractions of horse spleen ferritin (Fischbach et al., 1969). These X-ray diffraction patterns require the cubic morphology of the core to be in registration with the protein shell (but not the crystal structure), suggesting that the protein is acting as a template for the shape of the mineral core.

Our 3D reconstructions were not from fully loaded cores, and therefore the clear absence of density in the centre of the models might be expected. This is seen in the raw images and class averages of the hepatic ferritin (Fig. 3) and was also noted by Isaacson and Ohtsuki (1980) in both dark field STEM images and inelastic-elastic difference-based STEM images of horse spleen ferritin. A small angle X-ray scattering (SAXS) study on horse spleen ferritin, apoferritin and intermediate states suggested a bimodal distribution of ferritin cores of sizes 1.8 and 7.2 nm (Zipper, 1993; Zipper et al., 1993). This is consistent with our images of a core formed of several subunits not meeting in the centre (at intermediate iron loading) but which at some critical iron loading (<2000 atoms) might meet and appear to be a single large core in terms of morphology. A more recent study on horse spleen ferritin with progressive amounts of iron removed (Galvez et al., 2008) produced similar SAXS distance distribution functions to Zipper et al. (1993) albeit a different interpretation; Galvez et al., suggested that a core of constant diameter of ∼5.8 nm is maintained over a range of iron loadings (200–2200 iron atoms) and that iron is extracted from the centre of a defective polyphasic core. Whilst there is no evidence for a polyphasic nature to the hepatic cores imaged in this study, the subunit structure evidenced here (Fig. 3) is consistent with Galvez et al.’s identification of a core of constant diameter that has variable iron density within. Subunit structure suggests that the mineral structure of the cores imaged in this study is not likely to be uniformly crystalline, as is suggested for horse spleen ferritins (Cowley et al., 2000), but instead contains crystalline domains (or coherent scattering domains) surrounded by more disordered material. This is confirmed by the atomic resolution STEM-HAADF imaging (Fig. 2d) which is consistent with recent work showing that ferrihydrite is a single phase mineral composed of varying sizes of coherent scattering domains (Michel et al., 2007). The SAXS data suggesting a 1.8 nm ‘small’ subunit size in horse spleen ferritin (Zipper, 1993) and the lattice image presented in this study showing a crystalline subunit of similar size in hepatic ferritin (Fig. 2d) are both consistent with the coherent scattering domain size of ferrihydrite (2–5 nm). Core structure (with varying mineral domain size) could explain the clear appearance of regular substructure in defocused bright field TEM images of extracted ferritin both on amorphous carbon substrates or in vitreous ice (Massover and Adrian, 1986; Massover, 1993). Our identification of a subunit structure to the core at intermediate iron loading is compatible with early X-ray data (Harrison et al., 1967) that shows that the horse spleen ferritin core is ‘crystalline and is composed of either a single crystallite or in some molecules of a small number of crystallites’ (Harrison and Hoy, 1970). The numbers of low or high iron-loaded cores in our data are insufficient for a reliable reconstruction of cores at the extremes of iron loading, however, we have ongoing work to extract and isolate such cores for further investigation.

The combination of our core subunit reconstruction (Fig. 3e) with the additional high resolution information from the HAADF images (Fig. 2d) and the established detailed protein structure and function that is known for ferritin (Chasteen and Harrison, 1999), suggests how the protein shell may act as a template for the shape of the mineral core. Here we present a new schematic model for the core growth process (Fig. 4). It is generally accepted that Fe^2+^ ions travel into the ferritin central cavity through the eight hydrophilic three-fold symmetry channels (Ford et al., 1984) and oxidise at specific protein sites, forming the mineral cores beyond the channel exits. At the early stage of the core formation, ferritin may contain more than one crystal nucleus near the exit of each of the eight entry channels (Fig. 4a). As iron storage requirements in the cell increase, more Fe^2+^ ions are loaded into ferritin, deposit and then oxidise directly at the surface of the existing parts of the core (Fig. 4b) (Chasteen and Harrison, 1999). Once one of these nuclei reaches a critical size it will become thermodynamically stable, forming a core subunit which, due to its lower free energy, will compete for further incoming iron, possibly at the expense of other neighbouring nuclei (Hoy et al., 1974); eventually, a structure of eight connected lobes or subunits within the core will be formed (Fig. 4c compared with Fig. 4d). This is consistent with our HAADF results and the high surface area to volume ratio of such a structure would enable rapid acquisition and release of iron facilitating ready response to cellular iron cycling. Since the inner surface of the core subunit is less accessible to incoming iron than the outer surface, a low density centre or even a hole would be left in the centre of the mineralised core. The likelihood of completely filling the whole cavity would be low and our core iron loading distribution with only a low percentage of cores with iron content greater than 2500 atoms (Fig. 3c) supports such a model. Others have also noted that the more usual iron loading of ferritin is close to 2000 atoms (for ferritin in pathological tissue, Iancu, 1992 and for extracted horse spleen ferritin, DeSilva et al., 1993). A fully saturated ferritin molecule would contain 4500 iron atoms but a less than full core is advantageous in terms of iron turn-over given that iron-rich ferritins are more resistant to breakdown than iron-poor molecules (Drysdale and Munro, 1966). Therefore it seems unlikely that cytosolic ferritin molecules have a full complement of iron, the highest iron loadings would be more likely to be found in ferritin situated in the lysosomes.

The subunit surfaces provide ideal sites for the dynamic turn-over of iron which could facilitate rapid iron deposition and release on a ‘last-in first-out’ basis as suggested by Hoy et al. (1974). There is evidence that most of the phosphate groups present in extracted hepatic ferritin cores are bound to surface sites (Treffry and Harrison, 1978) implying that these play an important role in the biological stability of the core. Phosphorous is indeed detected by STEM energy dispersive X-ray (EDX) spectroscopy in our analysis (where on average cores contain an atomic ratio of ∼0.1 P to Fe, Pan, 2007) and its precise location will be investigated. Given that phosphorus is at least suggested to be a surface component of a ferritin core, one can infer from our model that phosphorus may preferentially bond to the loosely packed Fe^3+^ ions at the surface of the subunits (yellow circles in Fig. 4c) such that elevated phosphorus levels may inhibit further crystallisation within the subunits.

Nuclear magnetic resonance measurements show that the centre and surface of ferritin cores (including iron-reconstituted ferritin from horse spleen apoferritin and directly extracted horse spleen ferritin) are different: the surface of the core is magnetically disordered whilst the interior is ordered (Brooks et al., 1998). The characteristic structural difference observed here between the surface and interior of a core would affect both core formation and the process of iron reduction and release from ferritin. A monolayer of surface atoms on a 2–2.5 nm diameter subunit would constitute 33% of the total atomic content of a core and thus ferritin cores themselves could provide a significant source for a labile iron pool that is able to meet normal fluctuations in cellular iron cycling.

We have concentrated on human hepatic ferritin (which has a protein shell rich in L subunits) but our findings have implication across the whole body where the structure and function of the molecule’s shell is different to that in the liver (the ratio of H to L subunits varies) (Harrison and Arosio, 1996; Chasteen and Harrison, 1999) as well as likely resonances with non-mammalian ferritins. Understanding the stability and reactivity of the non-haem iron storage pool has important implications throughout biology, from informing on the acquisition of iron from non-haem dietary iron sources to better insights into iron-related contributions to diseases (Schenck and Zimmerman, 2004; Crichton and Ward, 2006).

## 5 Conclusions

Analytical scanning transmission electron microscopy has been successfully applied to the characterisation of cytosolic ferritin mineral cores in thin sections of tissue from a human liver biopsy. We have identified chemical and structural modification of the mineral cores due to exposure to the high energy electron beam by assessing the electron fluence. Subsequent high resolution HAADF imaging of the mineral core confirms the polycrystalline structure of the core and shows a surface structure that is disordered and not facetted. Quantitative EELS allows calibration of the contrast level in the HAADF images of cores and enables us to show the range and distribution of iron loading in 1241 individual cores. A 3D reconstruction of an ‘average’ core containing around 1500 iron atoms is presented which has been constructed from the projected images of 750 cores of similar iron loading in random orientations. This reconstruction provides strong evidence that the hepatic ferritin core has a regular subunit structure that reflects the cubic symmetry of the protein shell. We propose a growth model for such a core that is based around the eight channels in the protein shell that are known to deliver iron ions for storage and produces a maximum of eight subunits in the core. The high surface area of a core with subunit structure combined with a disordered or dynamic and potentially functionalised surface may help explain the ability of ferritin to rapidly turn-over iron when providing iron storage and suggests that ferritin itself could be a source for a labile pool of iron for rapid intracellular response to fluctuations in cellular iron cycling.
